# Supplementary material for: Impaired histone inheritance promotes tumor progression
Source: Nat Commun. 2023 Jun 10;14:3429. doi: 10.1038/s41467-023-39185-y (PMC10257670; doi:10.1038/s41467-023-39185-y)
Supplement: Supplementary file 1 — Supplementary Information [file 41467_2023_39185_MOESM1_ESM.pdf]

Supplementary Information

Impaired histone inheritance promotes tumor progression

Congcong Tian[1,#], Jiaqi Zhou[1,#], Xinran Li[1,#], Yuan Gao[2], Qing Wen[1], Xing Kang[1], NanWang[1], Yuan Yao[1], Jiuhang Jiang[1,3], Guibing Song[1,4], Tianjun Zhang[1,5], Suili Hu[1,3], JingYi Liao[1], Chuanhe Yu[6], Zhiquan Wang[7], Xiangyu Liu[8], Xinhai Pei[9], Kuiming Chan[10,11], Zichuan Liu[12], and Haiyun Gan[1,*]

Supplementary Figure 1

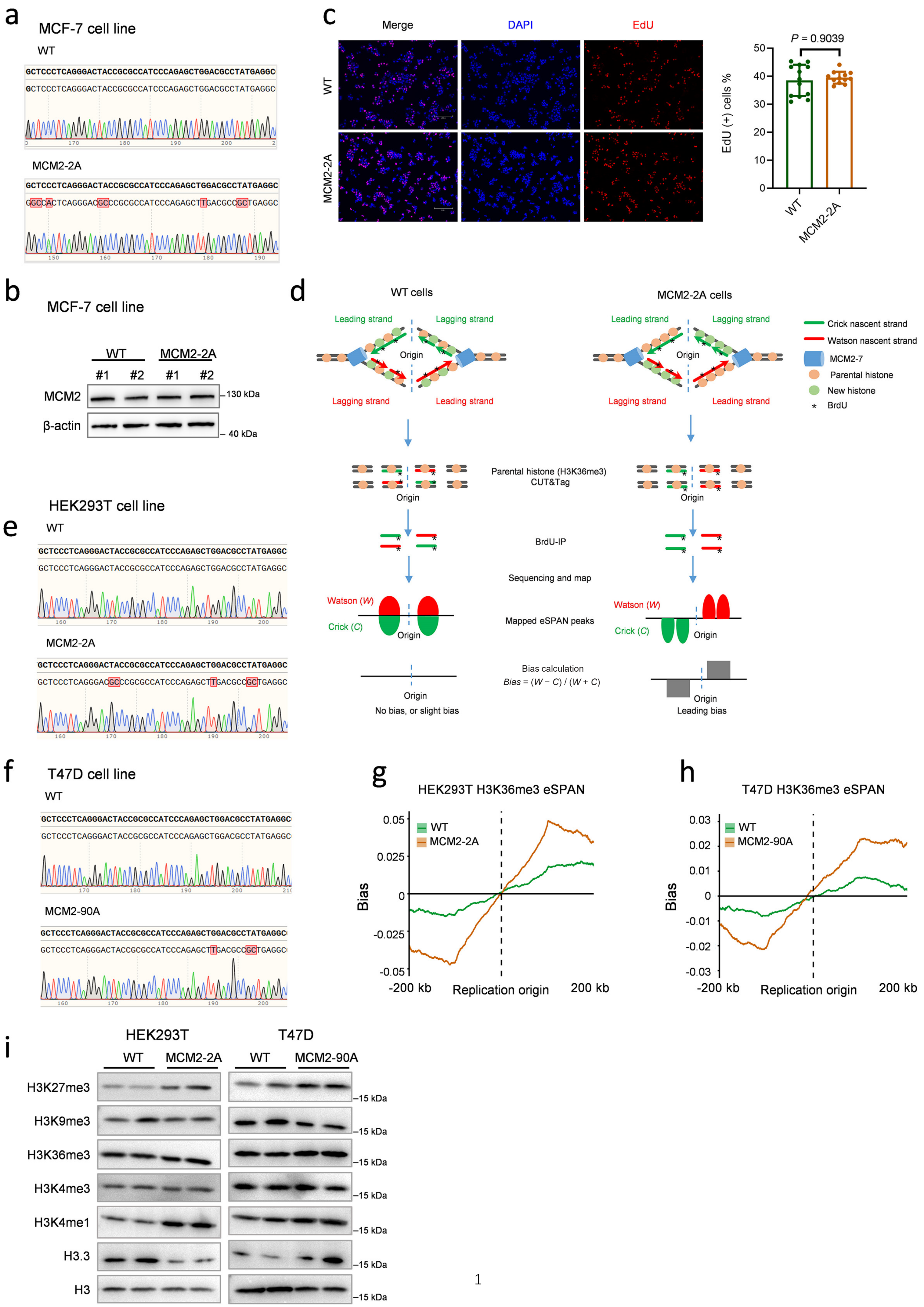

Supplementary Figure 1. Impaired parental histone inheritance in MCM2 mutant breast cancer and noncancerous cell lines.

a Sanger sequencing confirms homozygous MCM2-2A mutation of selected MCF-7 colony. **b** Immunoblots of MCM2 in MCM2-2A mutant and WT MCF-7 cells. β-actin was used as a loading control. This experiment was repeated 3 times independently with similar results. **c** EdU staining of MCM2-2A and WT MCF-7 cells. Cells were treated with EdU at 10 μM for 2 h. The number of EdU-positive cells and all the cells (marked by DAPI) was determined with ImageJ software. Data are presented as mean values ± SD (WT, n = 14 visions over 2 independent clones; MCM2-2A, n = 12 visions over 2 independent clones). Two-sided Mann-Whitney test. **d** H3K36me3 enrichment and sequencing of protein-associated nascent DNA (eSPAN) analysis. We analyzed the impact of MCM2-2A mutation on nucleosome assembly using surrogate marks of parental histones (H3K36me3) as reported previously.[1, 2] The bias of H3K36me3 eSPAN at selected replication origins (n = 7,624) was computed from unique fragments in each bin using the formula $Bias = (W - C) / (W + C)$; where W and C are the number of fragments mapped onto the Watson and Crick strands in each bin, respectively. There is no bias or a slight leading bias in WT cells, representing the parental histones are symmetrically recycled to the leading strands and the lagging strands. There is a leading bias in MCM2-2A mutant cells, which means parental histones recycled to the leading strands are more than that to the lagging strands. **e, f** Sanger sequencing confirming the homozygous MCM2-2A HEK293T colony (**e**) and homozygous MCM2-90A T47D colony (**f**). **g** Average strand bias of H3K36me3 in MCM2-2A mutant and WT HEK293T cells, as determined using eSPAN. **h** Average strand bias of H3K36me3 in MCM2-90A and WT T47D cells determined by eSPAN. **i** Western blot analysis showing select histone marks and histone variant H3.3 in MCM2-2A mutant and WT HEK293T cells, as well as in MCM2-90A and WT T47D cells. This experiment was repeated twice independently with similar results. EdU, 5-Ethynyl-2'-deoxyuridine; DAPI, 2-(4-Amidinophenyl)-6-indolecarbamidine dihydrochloride; BrdU, 5-Bromo-2'-deoxyuridine.

Supplementary Figure 2

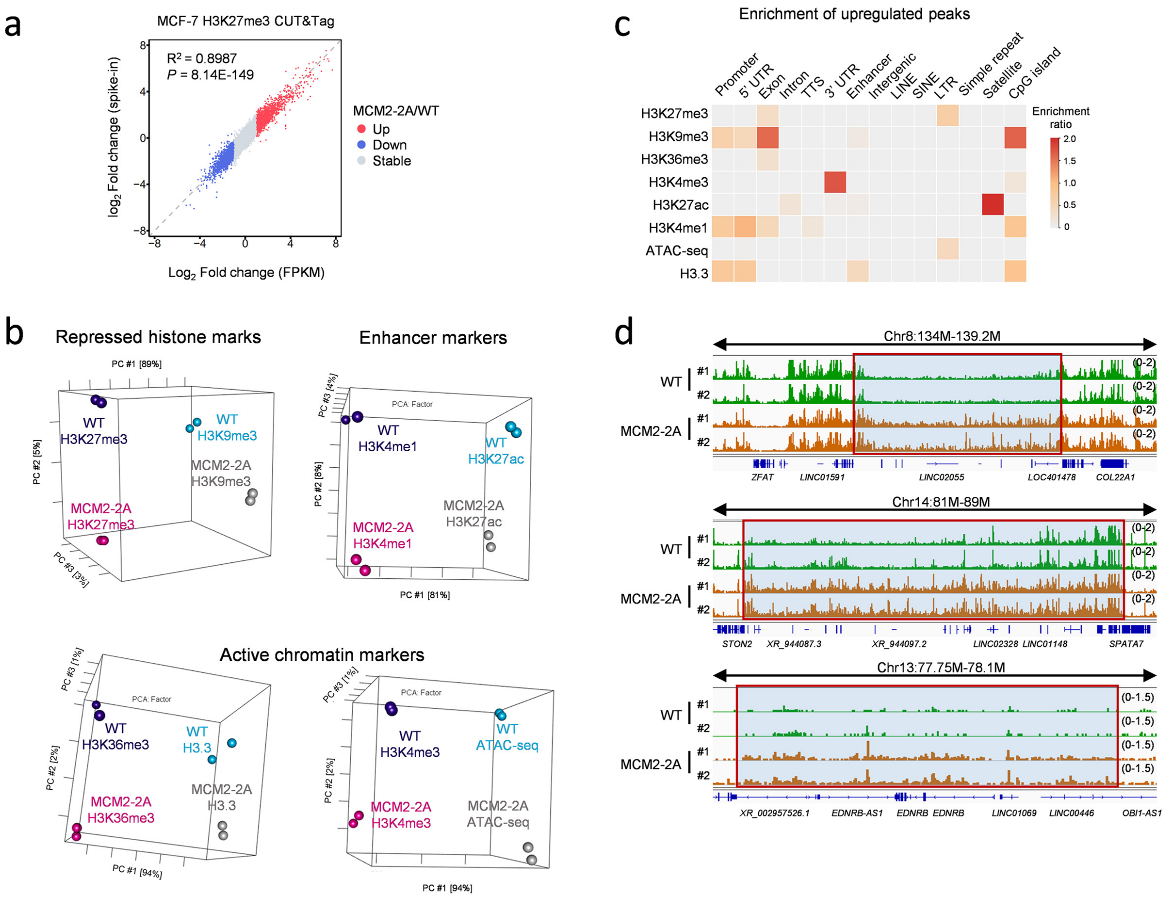

Supplementary Figure 2. MCM2-2A mutation alters epigenetic landscape of MCF-7 breast cancer cells.

a Dotplot showing the correlation of two normalization methods. *x*-axis shows the fold change of H3K27me3 in each bin following the normalization to library size (Fragments Per Kilobase per Million mapped fragments, FPKM), *y*-axis shows the fold change of H3K27me3 in each bin following the normalization to the spike-in genomic DNA derived from the *E. coli* during transposase protein production[3]. Two-sided Pearson correlation test suggests that the results from the two normalization methods are comparable (R^2 = 0.8987; *P* = 8.14E-149). **b** Principal component analysis (PCA) of the profiles of repressed histone marks (H3K27me3 and H3K9me3), enhancer markers (H3K4me1 and H3K27ac), and active chromatin markers (H3K4me3, H3K36me3, histone variant H3.3, as well as chromatin accessibility characterized using ATAC-seq) in MCM2-2A mutant and WT MCF-7 cells. **c** Integrative analysis showing which types of genomic regions are enriched for upregulated histone marks, histone variant H3.3 or chromatin accessibility (ATAC-seq) in MCM2-2A mutant MCF-7 cells. The color filled in each cell represents the enrichment ratio of the upregulated peaks superior to the stable peaks. The detailed calculation process of the enrichment ratio is depicted in Methods. **d** Integrative Genomics Viewer tracks showing the distribution of upregulated H3K27me3 at intergenic regions in MCM2-2A mutant and WT MCF-7 cells (red box). UTR, untranslated region; TTS, transcription termination sites; LINE, long interspersed nuclear elements; SINE, short interspersed nuclear elements; LTR, long terminal repeat retrotransposons; PC, principal component.

Supplementary Figure 3

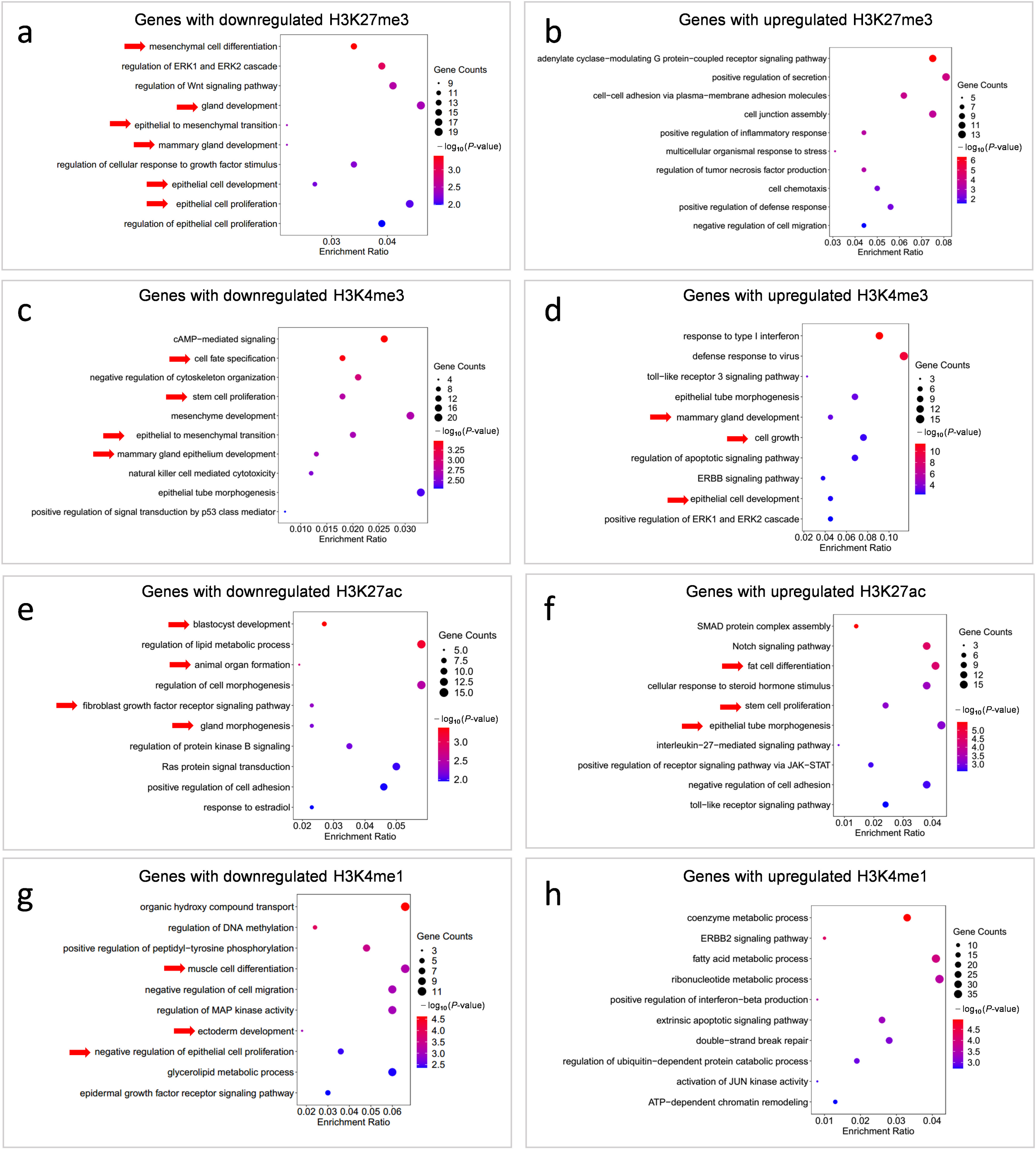

Supplementary Figure 3. GO analysis for the genes with altered histone mark peaks nearby in MCM2-2A mutant MCF-7 cells.

Gene Ontology (GO) terms enriched for genes associated with up- or downregulated H3K27me3 (**a** & **b**), H3K4me3 (**c** & **d**), H3K27ac (**e** & **f**), and H3K4me1 (**g** & **h**) peaks (within ± 2 kb TSS region) in MCM2-2A mutant *vs.* WT MCF-7 cells. One-sided hypergeometric test without adjustment was used to calculate statistical significance.

Supplementary Figure 4

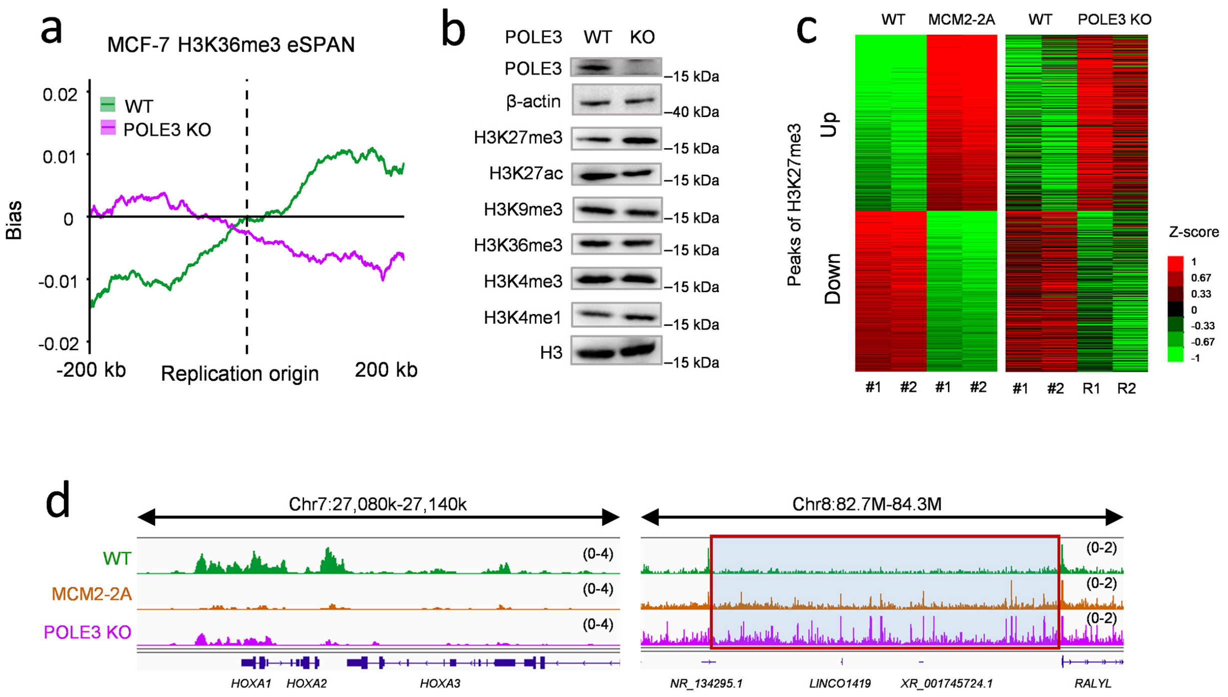

Supplementary Figure 4. POLE3 deletion results in similar H3K27me3 reprogramming as MCM2-2A mutation in MCF-7 cell line.

a Average bias of H3K36me3 in POLE3 KO and WT MCF-7 cells, as determined using eSPAN. The lagging bias in POLE3 KO cells means parental histone H3K36me3 recycled to the lagging strand is more than that recycled to the leading strand. **b** Western blot analysis of select histone marks using total cell lysates from POLE3 KO and WT MCF-7 cells. This experiment was repeated twice independently with similar results. **c** Heatmaps showing signal intensity changes of H3K27me3 levels in MCM2-2A mutant *vs.* WT cells (left) and corresponding signal changes of H3K27me3 level in POLE3 KO *vs.* WT cells (right). Alteration of H3K27me3 in POLE3 KO cells was consistent with the changes of H3K27me3 in MCM2-2A mutant cells. R1 and R2 indicates two replicates. #1 and #2 indicate two independent clones. **d** Integrative Genomics Viewer tracks showing the distribution of H3K27me3 at indicated loci in MCM2-2A mutant, POLE3 KO and WT cells. In both MCM2-2A mutant and POLE3 KO cells, H3K27me3 was downregulated at development-related *HOXA* cluster (left), while H3K27me3 were upregulated at randomly selected intergenic regions (right). eSPAN, enrichment and sequencing of protein-associated nascent DNA.

Supplementary Figure 5

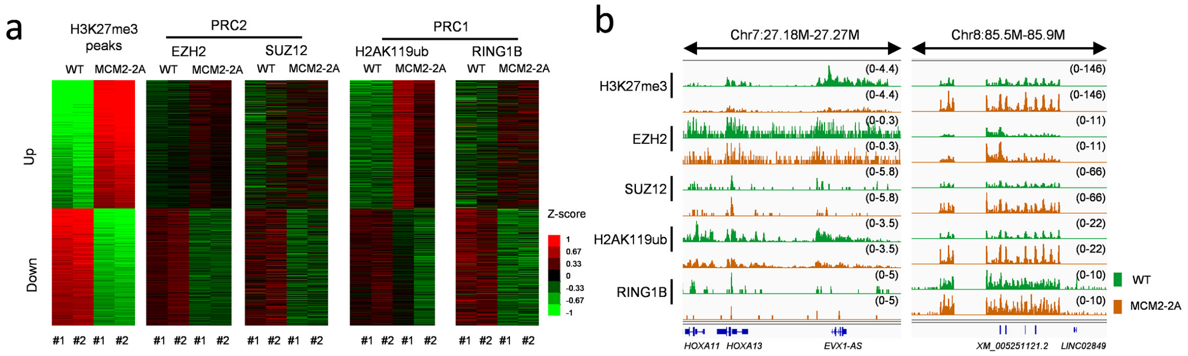

Supplementary Figure 5. PRC1/2 occupancies are correlated with H3K27me3 alteration in MCM2-2A mutant MCF-7 cells.

a Heatmaps showing signal intensity changes of H3K27me3 levels in MCM2-2A mutant *vs.* WT cells and corresponding signal of EZH2 (ChIP-seq data), SUZ12, H2AK119ub, and RING1B (CUT&Tag data) in MCM2-2A mutant and WT cells. Alteration of Polycomb repressive complex 2 (PRC2, including EZH2 and SUZ12) and Polycomb repressive complex 1 (PRC1, including H2AK119ub and RING1B) was correlated with the changes of H3K27me3. #1 and #2 indicate two independent clones. **b** Integrative Genomics Viewer tracks showing distribution of H3K27me3, EZH2, SUZ12, H2AK119ub, and RING1B at the indicated loci in MCM2-2A mutant and WT cells. Development-related *HOXA* cluster and randomly selected intergenic regions were picked as an example for (**a**).

Supplementary Figure 6

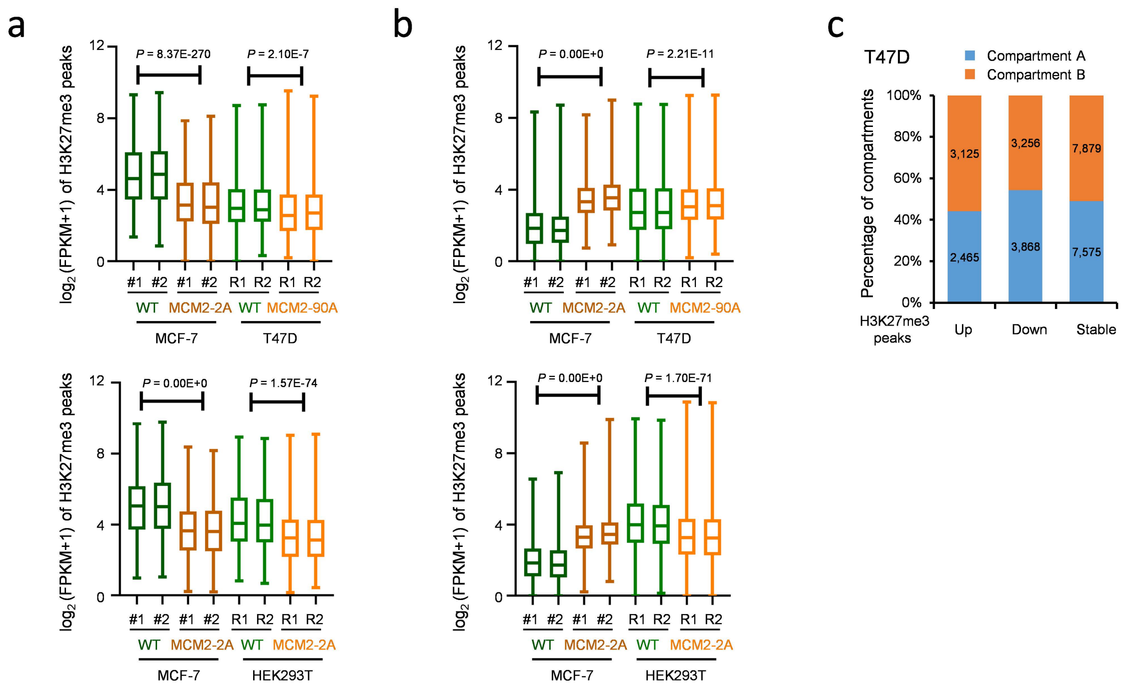

Supplementary Figure 6. The similarity of H3K27me3 reprogramming among MCM2 mutant line cells.

a, b Boxplot showing the H3K27me3 signal in indicated cells at H3K27me3 downregulated (n = 350 peaks overlapped in MCF-7 and T47D cells, n = 513 peaks overlapped in MCF-7 and HEK293T cells) (**a**) or upregulated peaks (n = 625 peaks overlapped in MCF-7 and T47D cells, n = 1,130 peaks overlapped in MCF-7 and HEK293T cells) (**b**) in MCM2-2A mutant MCF-7 cells. #1 and #2 indicate two independent clones, R1 and R2 indicate two replicates. The box plots display the median, upper and lower quartiles; the whiskers show 1.5× interquartile range (IQR). Two-sided paired *t*-test. **c** Proportion of H3K27me3 peaks belonging to A-type *vs.* B-type compartments, stratified by whether the peaks were upregulated, stable, or downregulated in MCM2-90A T47D cells. FPKM, Fragments Per Kilobase per Million mapped fragments.

Supplementary Figure 7

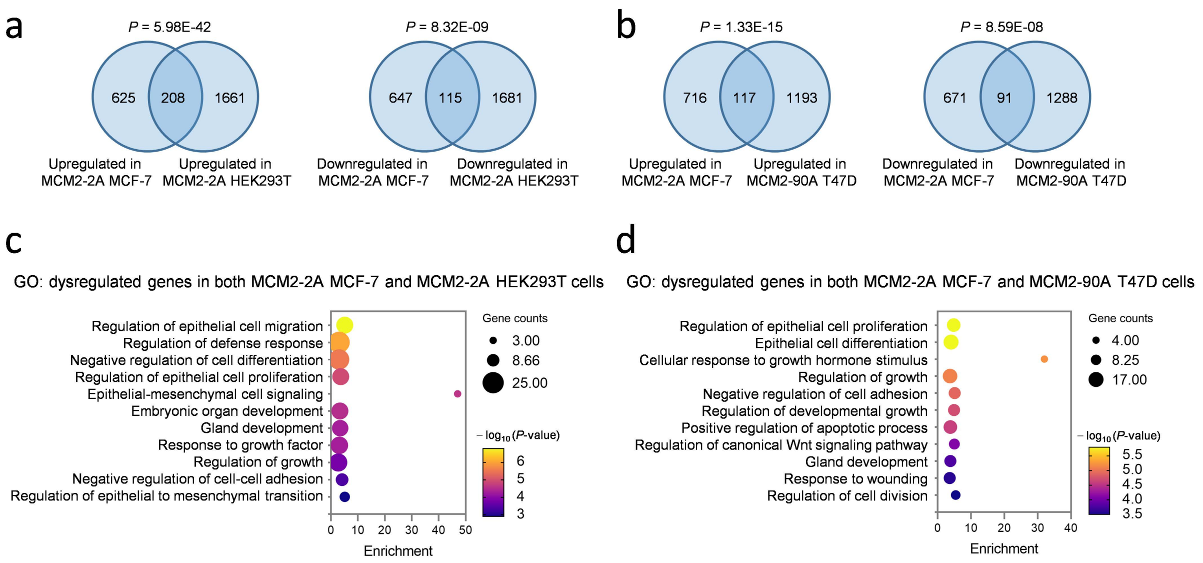

Supplementary Figure 7. The similarity of gene expression changes among MCM2 mutant cell lines.

a Venn plot showing the upregulated (left) or downregulated (right) genes (MCM2-2A/WT) in both MCM2-2A MCF-7 and MCM2-2A HEK293T cells compared to their WT counterparts. **b** Venn plot showing the upregulated (left) or downregulated (right) genes in both MCM2-2A MCF-7 and MCM2-90A T47D cells compared to their WT counterparts. Two-sided Chi-square test was performed to calculate statistical significance in (**a**, **b**). **c, d** Gene Ontology (GO) terms enriched for the dysregulated genes in both MCM2-2A MCF-7 and MCM2-2A HEK293T cells (**c**), as well as in both MCM2-2A MCF-7 and MCM2-90A T47D cells (**d**). One-sided hypergeometric test without adjustment was used to calculate statistical significance.

Supplementary Figure 8

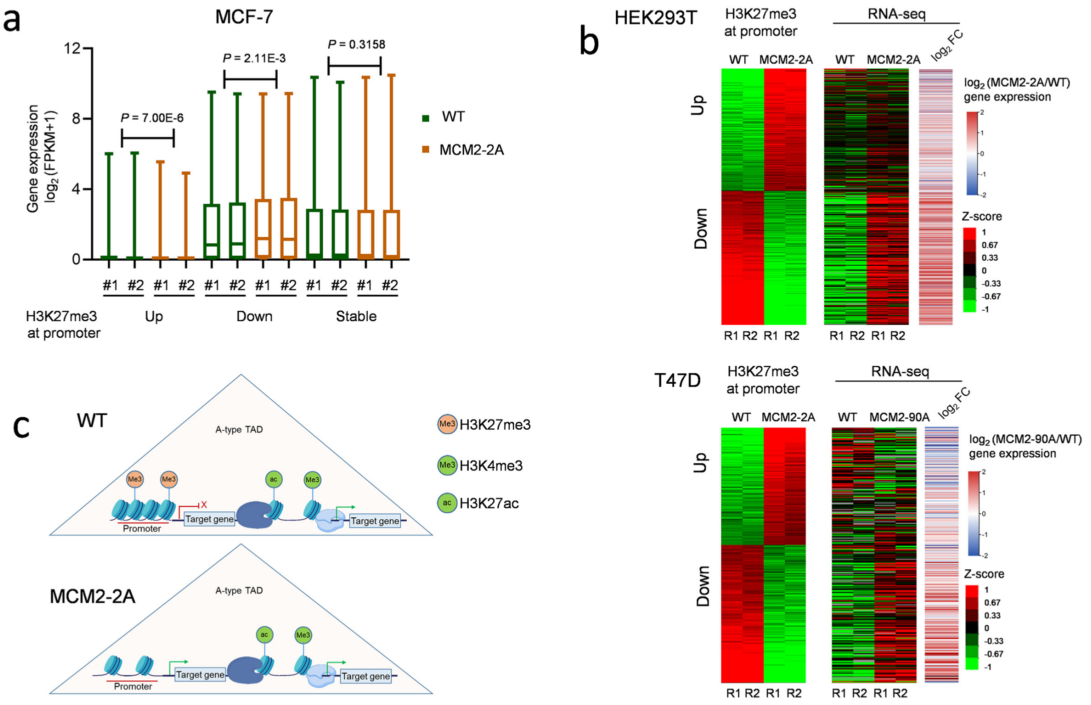

Supplementary Figure 8. Gene expressions are correlated with H3K27me3 alteration in

MCM2 mutant cell lines.

a Boxplots representing the expression levels of genes with promoters at which H3K27me3 was upregulated (n = 850), stable (n = 6,865), or downregulated (n = 2,297) in MCM2-2A mutant *vs.* WT MCF-7 cells. Two-sided paired *t*-test indicates that the expression of genes with H3K27me3 downregulated promoters increased significantly in MCM2-2A cells, whereas, those with H3K27me3 stable or upregulated promoters are low in both MCM2-2A and WT MCF-7 cells. #1 and #2 indicate two independent clones. The box plot displays the median, upper and lower quartiles; the whiskers show 1.5× IQR. **b** Heatmap showing signal intensity of H3K27me3 at promoters (left) and the expression of their target genes (right) in MCM2-2A mutant and WT HEK293T cells (upper), as well as in MCM2-90A and WT T47D cells (lower). R1 and R2 indicate two replicates. **c** Model showing impaired histone inheritance results in the downregulation of the repressive histone mark H3K27me3 at gene promoters in A-type compartments in MCM2-2A mutant cells, leading to greater expression of the corresponding genes. Created with BioRender.com. FPKM, Fragments Per Kilobase per Million mapped fragments; FC, fold change; TAD, Topological Associated Domains.

Supplementary Figure 9

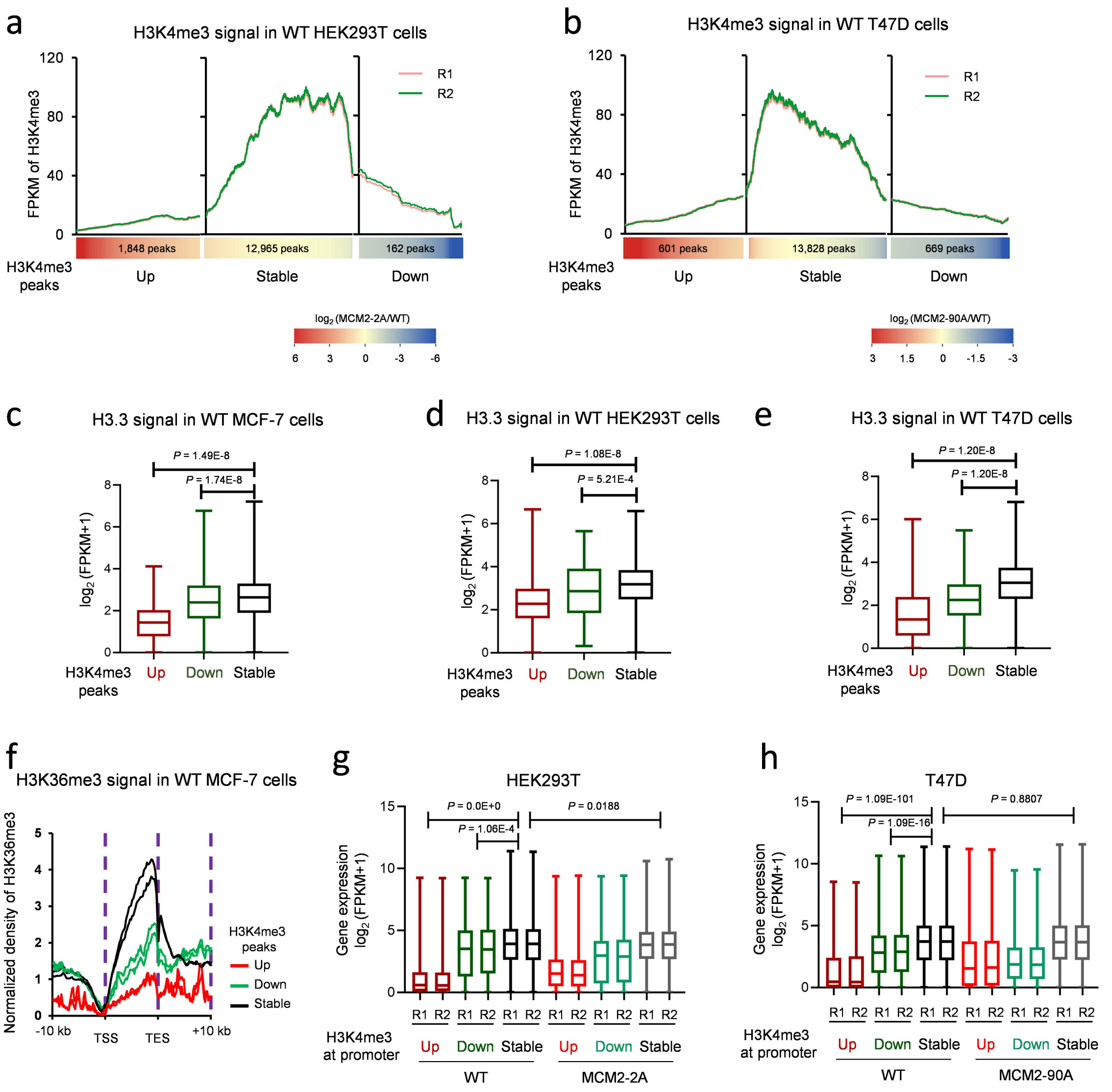

Supplementary Figure 9. H3K4me3 remains stable at more active chromatin regions in

MCM2 mutant cell lines.

a, b H3K4me3 signal in WT HEK293T cells (**a**) and WT T47D cells (**b**), at H3K4me3 peaks that were upregulated, stable, or downregulated in MCM2-2A mutant HEK293T cells and MCM2-90A T47D cells, respectively. R1 and R2 indicate two replicates. **c** Boxplots showing levels of the histone variant H3.3 in WT MCF-7 cells at regions where H3K4me3 was upregulated (n = 189), stable (n = 13,751), or downregulated (n = 1,446) in MCM2-2A mutant MCF-7 cells. **d** Boxplots showing H3.3 levels in WT HEK293T cells at peaks where H3K4me3 were upregulated (n = 1,848), stable (n = 12,965), or downregulated (n = 162) in MCM2-2A mutant HEK293T cells. **e** Boxplots showing H3.3 levels in WT T47D cells at peaks where H3K4me3 were upregulated (n = 601), stable (n = 13,828), or downregulated (n = 669) in MCM2-90A T47D cells. One-way ANOVA adjusted by Tukey's multiple comparisons test was used to calculate statistical significance in (**c-e**). **f** Average signal of H3K36me3 at gene bodies [from 10 kb upstream of the transcription start site (TSS) to 10 kb downstream of the transcription end site (TES)] whose H3K4me3 was upregulated, stable, or downregulated in MCM2-2A mutant MCF-7 cells. Two independent WT MCF-7 clones were shown. **g** Boxplots representing expression level of genes with H3K4me3 upregulated (n = 1,531), stable (n = 8,724), or downregulated (n = 263) promoters in MCM2-2A mutant (right) and WT (left) HEK293T cells. **h** Boxplots representing expression level of genes with H3K4me3 upregulated (n = 405), stable (n = 19,270), or downregulated (n = 568) promoters in MCM2-90A (right) and WT (left) T47D cells. Two-way repeated measures ANOVA adjusted by LSD for multiple comparisons was used to calculate statistical significance in (**g**, **h**). The box plots in (**c-e** and **g-h**) display the median, upper and lower quartiles; the whiskers show 1.5× IQR. FPKM, Fragments Per Kilobase per Million mapped fragments; TSS, transcriptional start site; TES, transcription end site.

Supplementary Figure 10

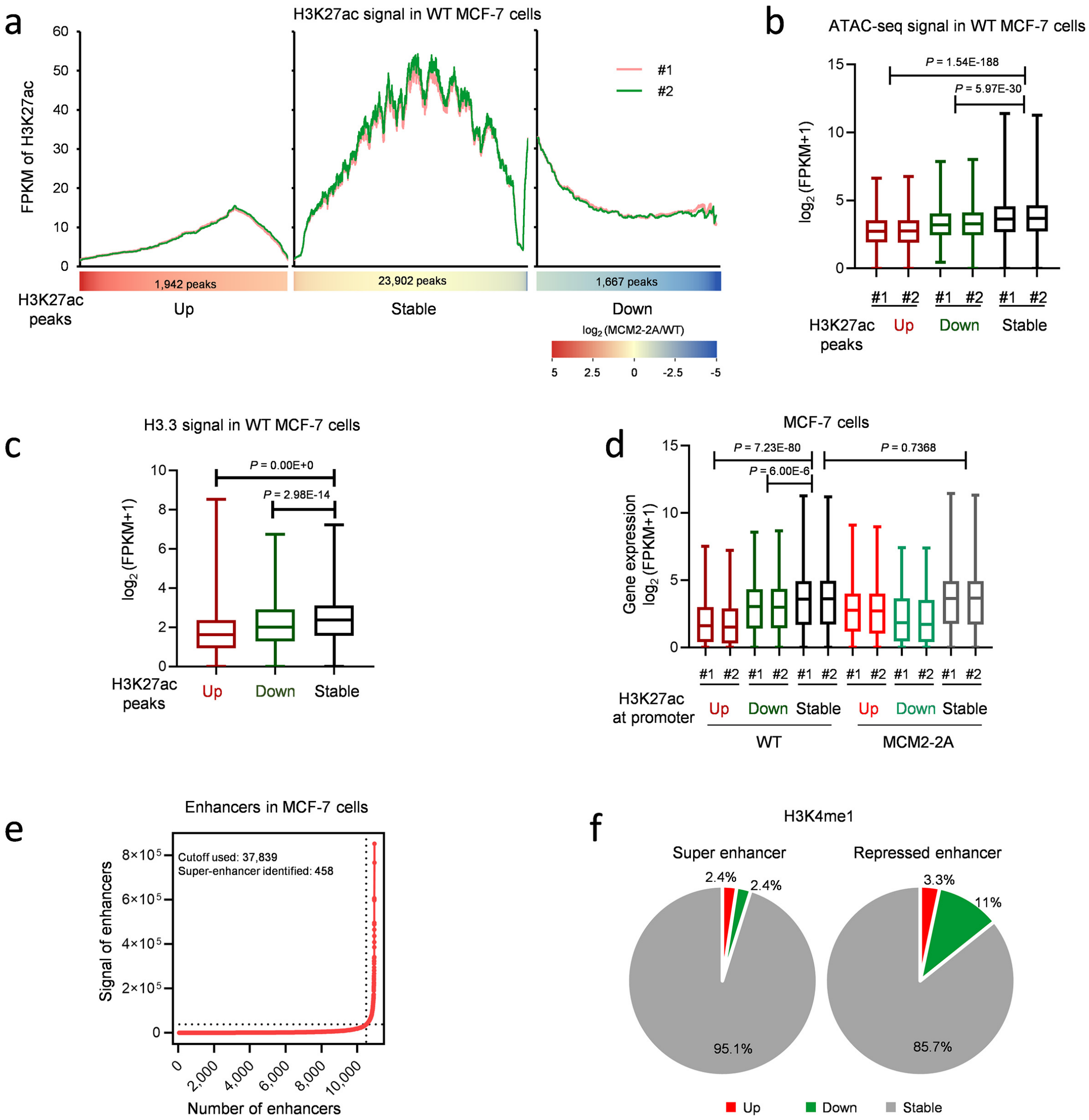

Supplementary Figure 10. In MCM2-2A mutant MCF-7 cells, transcriptionally active chromatin state remains stable during epigenetic reprogramming.

a H3K27ac signal in WT MCF-7 cells, at H3K27ac peaks that were upregulated, stable, or downregulated in MCM2-2A mutant MCF-7 cells. #1 and #2 indicate two independent WT MCF-7 clones. **b** Boxplots depicting chromatin accessibility (ATAC-seq signal) in WT MCF-7 cells, at H3K27ac peaks that were upregulated (n = 1,942), stable (n = 23,902), or downregulated (n = 1,667) in MCM2-2A mutant MCF-7 cells. Two-way repeated measures ANOVA adjusted by LSD for multiple comparisons was used to calculate statistical significance. **c** Boxplots depicting H3.3 levels in WT MCF-7 cells, at H3K27ac peaks that were upregulated (n = 1,942), stable (n = 23,902), or downregulated (n = 1,667) in MCM2-2A mutant MCF-7 cells. One-way ANOVA adjusted by Tukey's multiple comparisons test. **d** Boxplots depicting expression of genes with H3K27ac upregulated (n = 688), stable (n = 20,199), or downregulated (n = 543) promoters in MCM2-2A mutant cells, for both MCM2-2A mutant (right) and WT (left) MCF-7 cells. Two-way repeated measures ANOVA adjusted by LSD for multiple comparisons was used to calculate statistical

significance. The box plots in (**b-d**) display the median, upper and lower quartiles; the whiskers show 1.5× IQR. **e** Cutoff value for identifying super-enhancers using ROSE scripts. **f** Proportion of peaks at which H3K4me1 was upregulated, stable, or downregulated in MCM2-2A mutant *vs.* WT MCF-7 cells, for super-enhancers (left) and repressed enhancers (right). FPKM, Fragments Per Kilobase per Million mapped fragments.

Supplementary Figure 11

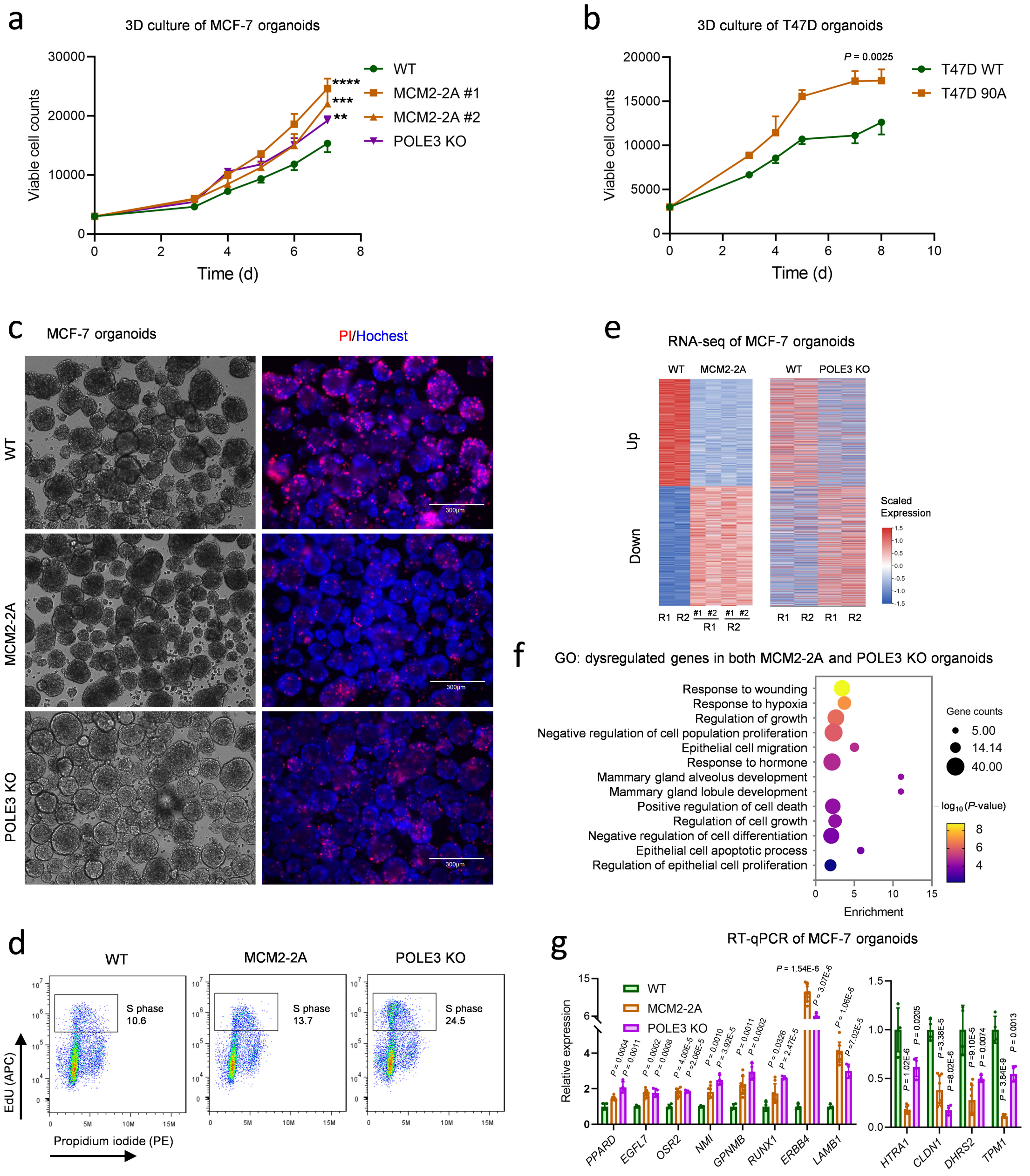

Supplementary Figure 11. Impaired histone inheritance promotes the tumor growth in MCF-7 and T47D organoids.

a The growth curve of the MCM2-2A mutant, POLE3 KO and WT MCF-7 organoids. #1 and #2 indicate two independent MCM2-2A mutant clones. Data are presented as mean values ± SD (n = 4 independent experiments for each clone). ****, ***, and ** represent $P = 7.49 \times 10^{-6}$, 1.23×10^{-4} and 0.0047, respectively. One-way ANOVA adjusted by Holm multiple comparisons test. **b** The growth curve of the MCM2-90A and WT T47D organoids. Data are presented as mean values ± SD

(n = 4 independent experiments for each genotype). Student’s *t*-test. **c** Images showing the morphology and cell alive state of MCM2-2A mutant, POLE3 KO and WT MCF-7 organoids. Propidium iodide, PI (+) represents dead cells, Hoechst (+) represents alive cells. This experiment was repeated twice independently with similar results. **d** Cell proliferation evaluated by EdU staining in MCM2-2A mutant, POLE3 KO and WT MCF-7 organoids. **e** Heatmap showing the differentially expressed genes in MCM2-2A mutant *vs.* WT MCF-7 organoids and their corresponding expression in POLE3 KO and WT MCF-7 organoids. #1 and #2 indicate two independent clones. R1 and R2 indicate two replicates. **f** Gene Ontology (GO) enrichment for the dysregulated genes in both MCM2-2A mutant and POLE3 KO MCF-7 organoids. One-sided hypergeometric test without adjustment was used to calculate statistical significance. **g** RT-qPCR showing the expression of proliferation-related genes in MCM2-2A mutant, POLE3 KO and WT MCF-7 organoids. The genes’ upregulation (left) or downregulation (right) could promote cancer cell proliferation. Data are presented as mean values ± SD. WT and POLE3 KO, n = 4 independent experiments; MCM2-2A, n = 8 (4 experiments over 2 independent clones). Two-sided Student’s *t*-test. qPCR, quantitative real-time PCR; RT, reverse transcription; EdU, 5-Ethynyl-2'-deoxyuridine.

Supplementary Figure 12

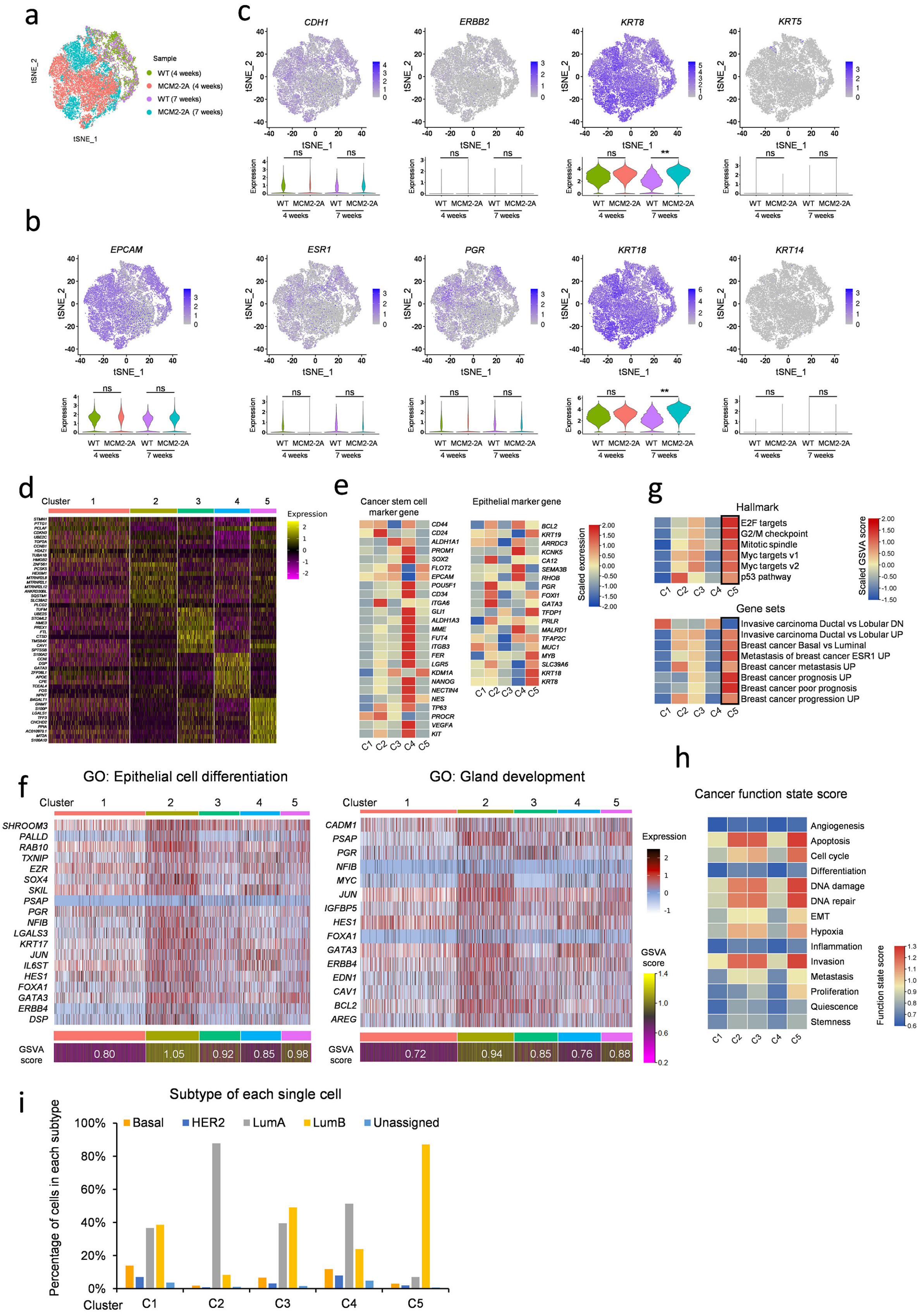

Supplementary Figure 12. Basic characteristics of MCM2-2A mutant and WT MCF-7 tumors at the single-cell level.

a *t*-SNE plot showing distribution of MCM2-2A mutant and WT tumor cells, as identified by

scRNA-seq data, harvested 4 and 7 weeks post orthotopic transplantation. **b**, **c** Expression of key breast cancer markers in each tumor cell on a *t*-SNE plot (upper) and a violin plot (lower). Cells are colored blue on *t*-SNE plots if a specific marker is expressed, with the intensity representing the gene expression level. Violin plots showing the expression levels of key breast cancer marker genes in MCM2-2A mutant and WT tumor cells harvested 4 and 7 weeks post-transplantation. The significance of differences was examined by two-sided Wilcoxon rank sum test. ** represents $P <$ 0.01 and absolute $\log_{10}$ (fold change) > 0.3; ns represents $P > 0.05$ or absolute $\log_{10}$ (fold change) < 0.3. The exactly value of *P* value and fold change was provided in the Source Data file. **d** Gene expression profile for each cluster's top 10 marker genes in each single cell. **e** Heatmaps showing average expression of cancer stem cell marker genes (left) and epithelial marker genes (right) for each cluster. **f** Heatmaps showing expression profile in each cluster for genes enriched in the gene ontology terms epithelial cell differentiation (left) and gland development (right). GSVA scores are shown below the heatmaps. Each column represents a single cell. **g** Heatmap showing the average GSVA score of cell proliferation-related hallmark pathways (upper) and breast cancer invasion-, metastasis- and progression-related gene sets (lower) for each cluster. The gene sets related to breast cancer invasion, metastasis and progression in patients are obtained from the GSEA website (https://www.gsea-msigdb.org/gsea/msigdb/genesets.jsp?collection=CGP). The color filled in each cell represents the average GSVA score for the gene sets in that cluster. **h** Heatmap showing the average function state score of cells in each cluster. The information of function state of breast cancer single cells was obtained from CancerSEA[4] (http://biocc.hrbmu.edu.cn/CancerSEA/). **i** Proportion of single cells assigned to each breast cancer subtype. *t*-SNE, *t*-distributed stochastic neighbor embedding; GSVA, Gene set variation analysis; GO, Gene Ontology; EMT, epithelial to mesenchymal transition; Basal, basal-like; HER2, HER2-enriched; LumA, luminal A; LumB, luminal B.

Supplementary Figure 13

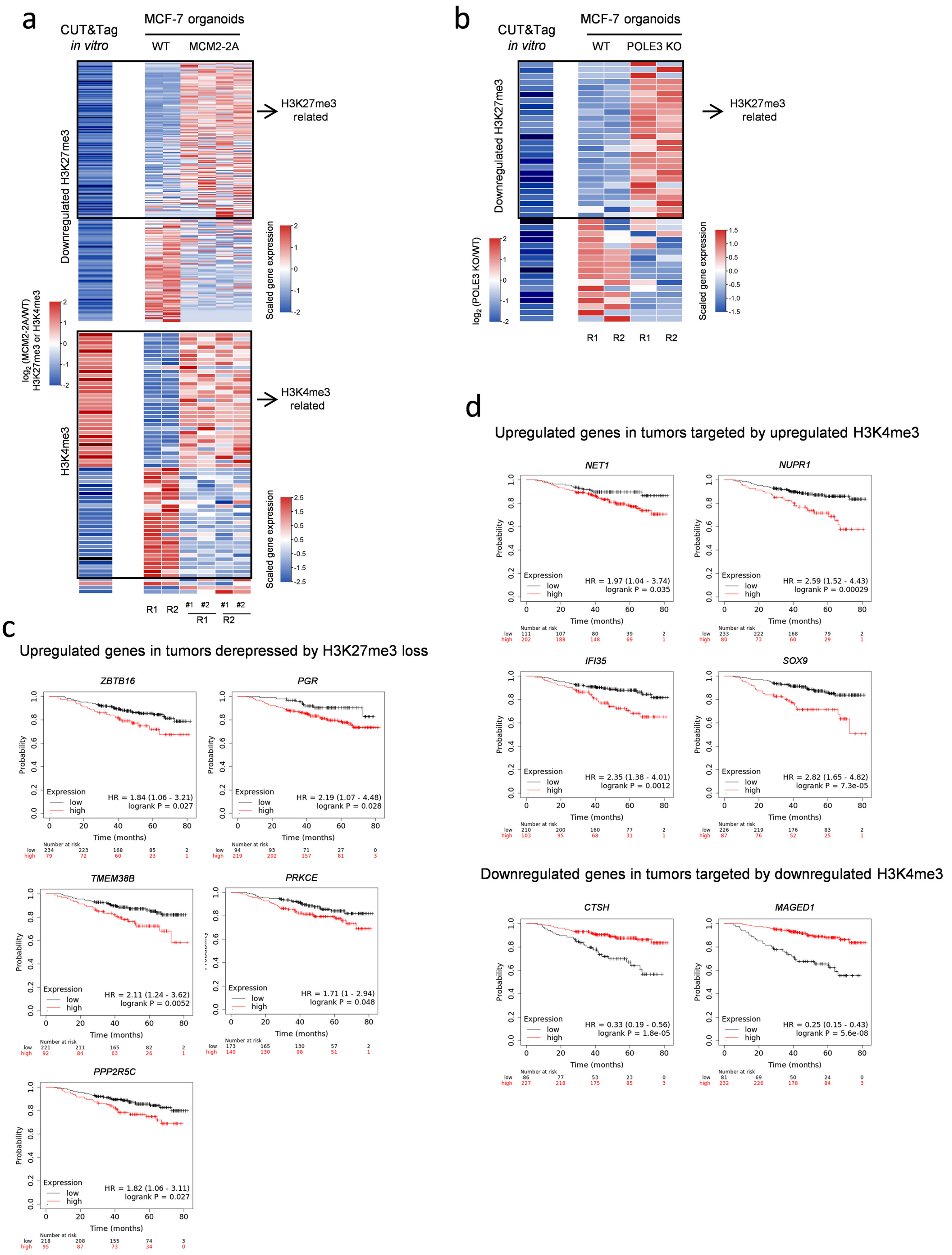

Supplementary Figure 13. Dysregulated histone marks affect the expression of proliferation-related genes in organoids and are associated with patient prognosis.

a Heatmap showing the fold change of CUT&Tag signal at H3K27me3 downregulated (upper) or H3K4me3 dysregulated (lower) promoters whose target genes are related to tumor growth, proliferation or metastasis, and their target genes' expression in MCM2-2A and WT MCF-7 organoids. #1 and #2 indicate two independent clones. R1 and R2 indicate two replicates. **b** Heatmap showing the fold change (POLE3 KO/WT) of H3K27me3 signal at H3K27me3 downregulated

promoters whose target genes are related to tumor growth, proliferation or metastasis, and their target genes' expression in POLE3 KO and WT MCF-7 organoids. **c, d** Kaplan–Meier survival analysis of breast cancer patients without endocrine- or chemo-treatment stratified by the expression of selected genes. The dysregulated genes in tumors derepressed by H3K27me3 loss (**c**) or targeted by dysregulated H3K4me3 (**d**) are associated with poor prognosis in breast cancer patients. The correlation between gene expression and survival in breast cancer patients was analysed by the Kaplan-Meier plotter online (http://kmplot.com/analysis/). HR, hazard ratio.

Supplementary Figure 14

a

Dominant clones' location

Dominant clone location (number)		C1	C2	C3	C4	C5
4 weeks	WT	0	0	10	0	0
	MCM2-2A	32	37	0	0	2
7 weeks	WT	0	0	13	0	0
	MCM2-2A	0	39	0	1	6

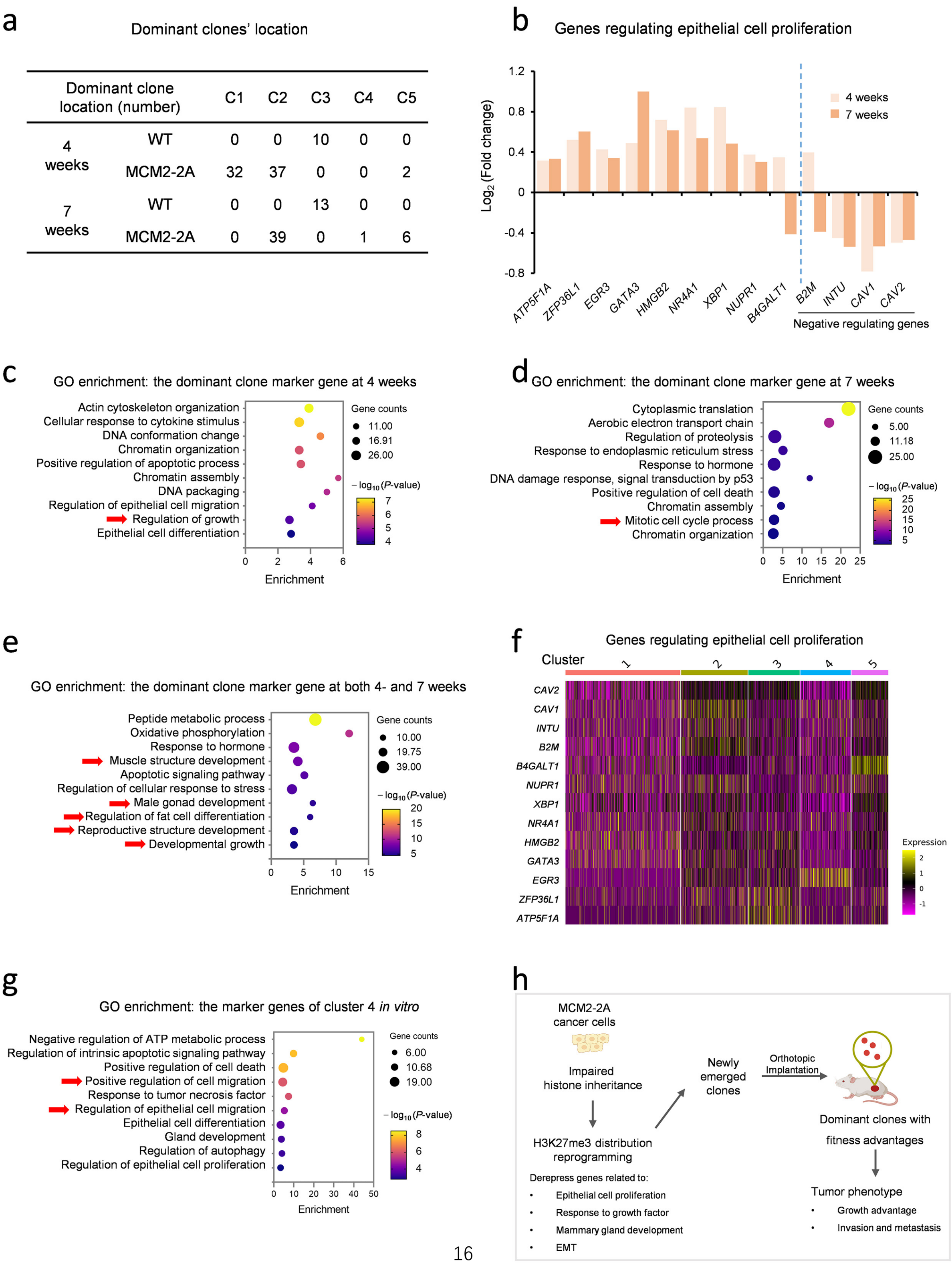

Supplementary Figure 14. Impaired histone inheritance confers dominant clones growth advantages *in vivo*.

a The number of dominant clones belonged to each cluster in MCM2-2A mutant and WT MCF-7 tumors. We defined the dominant clone belong to a cluster when the majority of cells in that dominant clone belong to that cluster. Cells in MCM2-2A mutant dominant clones mainly located within cluster 2, especially at 7 weeks post-transplantation. **b** Bar graph showing that genes related to regulation of epithelial cell proliferation displayed dramatic changes in MCM2-2A mutant dominant clones *vs.* all WT tumor cells. The significance of differences was examined by the Wilcoxon rank-sum test. **c-e** Gene ontology (GO) enrichment analysis for marker genes in dominant clones identified (**c**) only at 4 weeks post-transplantation, (**d**) only at 7 weeks post-transplantation, and (**e**) both at 4 weeks and 7 weeks post-transplantation. One-sided hypergeometric test without adjustment was used to calculate statistical significance. **f** Heatmap showing expression of genes listed in (**b**). Cells in cluster 2 from tumor tissues expressed highest levels of these genes. Each column represents a single tumor cell. **g** Gene ontology enrichment analysis for marker genes of cluster 4 *in vitro*. One-sided hypergeometric test without adjustment was used to calculate statistical significance. **h** Model of tumor progression driven by impaired histone inheritance in MCM2-2A mutant cancer cells. Created with BioRender.com. EMT, epithelial to mesenchymal transition.

Supplementary Figure 15

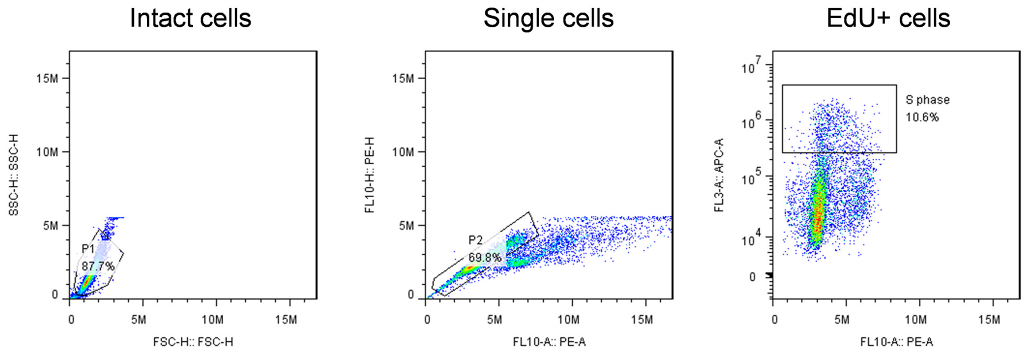

Supplementary Figure 15. Gating strategy for FACS.

Percentage of EdU positive cells was analyzed by flow cytometry. This gating strategy has been applied to generate the data in Supplementary Fig. 11d. Cells in WT, MCM2-2A and POLE3 KO MCF-7 organoids were labeled with PI (PE) and EdU (APC). Intact cells were selected by comparing cell size (FSC-A) and cell granularity (SSC-A). Single cells were detected by analyzing the PI cell area versus the PI height. EdU + cells were selected according to the APC signal. PI, Propidium iodide; EdU, 5-Ethynyl-2'-deoxyuridine.

Supplementary Table 1. Annotations for H3K27me3 peaks upregulated, stable, or downregulated in MCM2-2A mutant *vs.* WT MCF-7 cells

	Number of peaks		
Annotation	Upregulated	Downregulated	Stable
3' UTR	16	29	88
TTS	23	49	143
LINE	761	539	2,800
SINE	318	386	1,730
Exon	73	84	264
Intron	736	848	4,530
Intergenic	1,122	947	5,442
Promoter	98	179	563
5' UTR	7	20	59
CpG island	22	80	188
LTR	619	274	1,788
Simple repeat	37	53	236
Satellite	21	16	64

Supplementary Table 2. Number of peaks with upregulated, stable, or downregulated histone marks, chromatin accessibility (ATAC-seq) and histone variant H3.3 in MCM2 mutant cells

		Number of peaks		
Cell line	Experiment	Upregulated	Downregulated	Stable
MCF-7 MCM2-2A	ATAC-seq	5,629	7,641	85,663
	H3K4me1 CUT&Tag	3,162	3,260	57,897
	H3K4me3 CUT&Tag	189	1,446	13,751
	H3K9me3 CUT&Tag	3,152	9,621	42,137
	H3K27ac CUT&Tag	1,942	1,667	23,902
	H3K27me3 CUT&Tag	3,989	3,639	18,602
	H3K36me2 ChIP-seq	299	31	4,095
	H3K36me3 CUT&Tag	372	313	24,731
	H3.3 CUT&Tag	2,071	544	44,147
HEK293T MCM2-2A	H3K4me3 CUT&Tag	1,848	162	12,965
	H3K27me3 CUT&Tag	6,298	6,506	19,330
T47D MCM2-90A	H3K4me3 CUT&Tag	601	669	13,828
	H3K27me3 CUT&Tag	5,590	7,124	15,456

Supplementary Table 3. Number and size of clones detected *in vivo* with scRNA-seq

	MCM2-2A 4 weeks	WT 4 weeks	MCM2-2A 7 weeks	WT 7 weeks	Total
Total clone number	768	626	219	112	1,725
Dominant clone number	71	10	45	13	139
% dominant clone	9.24%	1.60%	20.55%	11.61%	8%
Max clone size	226	124	949	251	
Cell number in dominant clones	3,273	408	4,783	901	

Supplementary Table 4. Oligos used in this study

Name	Experiment	Sequence
H-MCM2-gRNA	Genome editing	GATGGCGCGGTAGTCCCTGA for MCF-7<br>CTACCGCGCCATCCCAGAGC for T47D and HEK293T
MCM2-2A SSODN	Genome editing	AGGGTTTTCCTTTTTGGCAGTAACCACATCTGTTTT<br>GGTGGCCACTCAGGGACGCCCGCGCCATCCCAGA<br>GCTTGACGCCGCTGAGGCCGAGGGACTGGCTCTG<br>GATGATGAGGACGTAGAGG
H-POLE3-gRNA	Genome editing	TGGAGATGTTGACACCGTCC

Supplementary Table 5. Primer pairs used for RT-qPCR in this study

Genes	Primer Forward (5'-3')	Primer Reverse (5'-3')
TGFB1	CAATTCCTGGCGATACCTCAG	GCACAACTCCGGTGACATCAA
STAT6	GTTCCGCCACTTGCCAATG	TGGATCTCCCCTACTCGGTG
AKAP12	GAGATGGCTACTAAGTCAGCGG	CAGTGGGTTGTGTTAGCTCTTC
LAMB1	AGGAACCCGAGTTCAGCTAC	CACGTCGAGGTCACCGAAAG
PGR	ACCCGCCCTATCTCAACTACC	AGGACACCATAATGACAGCCT
ANXA3	TTAGCCCATCAGTGGATGCTG	CTGTGCATTTGACCTCTCAGT
CXCL12	ATTCTCAACACTCCAAACTGTGC	ACTTTAGCTTCGGGTCAATGC
PRKCE	CGAGGCCGTGAGCTTGAAG	GCAATGTAGGGGTCGAGAAGG
IL6ST	CGGACAGCTTGAACAGAATGT	ACCATCCCACTCACACCTCA
FKBP1A	CTCCAGATTATGCCTATGGTGC	AGCTCCACATCGAAGACGAGA
ERBB4	GTCCAGCCCAGCGATTCTC	AGAGCCACTAACACGTAGCCT
PPARD	CAGGGCTGACTGCAAACGA	CTGCCACAATGTCTCGATGTC
EGFL7	TGAATGCAGTGCTAGGAGGG	GCACACAGAGTGTACCGTCT
OSR2	TCCGCCTAAGATGGGAGACC	GGTAAAGTGTCTGCCGCAAAA
NMI	AAGGAGCATTCGCCAGATGAA	GTAGCCTCTTGTAACTCCGTTTC
GPNMB	AAGATTGCCACTTGATGCCG	TCCCTCATGTAAGCAGAAGGTC
RUNX1	CTGCCCATCGCTTTCAAGGT	GCCGAGTAGTTTTCATCATTGCC
HTRA1	TCCCAACAGTTTGCGCCATAA	CCGGCACCTCTCGTTTAGAAA
CLDN1	CCTCCTGGGAGTGATAGCAAT	GGCAACTAAAATAGCCAGACCT
DHRS2	CCTCTGGTAGGGAGCACTCT	CCAGCGCCACTACTGGATTA
TPM1	GCCGACGTAGCTTCTCTGAAC	TTTGGGCTCGACTCTCAATGA

Supplementary Table 6. Primers used for H3K27me3 CUT&Tag qPCR

Regions	Primer Forward (5'-3')	Primer Reverse (5'-3')
TACR1	GCAATGCCGTGGTCCTCTAT	ACGGCCTGTTCTACTGCAAG
ETV4	TGGGGCCAAGTTCCATTCAA	CACCCAGCGTGGACACTTAT
TGFA	AGCATCCGGTTCCAAGACTG	TTCGTTCGATATGCCCCGAG
TGFB1	AAGCGGTCCACTTCGCTATC	GTGTCCGAAAGAGGATGGCA
BCL11B	TACTTACCCAGCTCCCCGTA	TCTCCCAAGTCGACCAACTG
STAT6	GGGCCTCTCAGTTCACGTTT	GAGAGGTGAGAGGCTGAACG
AKAP12	CCGTTTAGATCCAATGCGCC	CAGAGTCCCGGGAGGGTTTA
LAMB1	AAAGAGGTTGGTGACGGGAC	TCTCAGCGCTTTCAGGCTAC
PGR	GGCAATTTAGTGACACGCGG	GGGTTTTAGTGAGGGGGCAG
ANXA3	GGATCCACTTAGCCATCTCGG	CAGGATGGGTGCGATTCCAA
CXCL12	GGCTGCAAGAGCACTCAGA	GCTGAAGAGAAACTCGCTGC
PRKCE	CCTAGCCCTGTGTGATGTGG	TCCAGAGAAGGTCCCACCAA
IL6ST	GTCGCCTCTCCCAGACTAGA	GAACATTCCGCTCTTTCCGC
FKBP1A	TTCCGTTTTCCCTGAAGCCT	AGATGGCCACTGTCTCAAAGG
ERBB4	GACTCCGGCCAATAGCAAGT	TTGGCACACTTGAGCCAGAA

Supplementary References

1. Li Z*, et al.* DNA polymerase alpha interacts with H3-H4 and facilitates the transfer of parental histones to lagging strands. *Sci Adv* **6**, eabb5820 (2020).

2. Xu X, Duan S, Hua X, Li Z, He R, Zhang Z. Stable inheritance of H3.3-containing nucleosomes during mitotic cell divisions. *Nat Commun* **13**, 2514 (2022).

3. Kaya-Okur HS*, et al.* CUT&Tag for efficient epigenomic profiling of small samples and single cells. *Nat Commun* **10**, 1930 (2019).

4. Yuan H*, et al.* CancerSEA: a cancer single-cell state atlas. *Nucleic Acids Res* **47**, D900-D908 (2019).
