# Supplementary material for: Impaired histone inheritance promotes tumor progression
Source: Nat Commun. 2023 Jun 10;14:3429. doi: 10.1038/s41467-023-39185-y (PMC10257670; doi:10.1038/s41467-023-39185-y)
Supplement: Supplementary file 3 — Reporting Summary [file 41467_2023_39185_MOESM3_ESM.pdf]

Corresponding author(s): Haiyun Gan

Last updated by author(s): 2023/5/13

Reporting Summary

Nature Portfolio wishes to improve the reproducibility of the work that we publish. This form provides structure for consistency and transparency in reporting. For further information on Nature Portfolio policies, see our Editorial Policies and the Editorial Policy Checklist.

Statistics

For all statistical analyses, confirm that the following items are present in the figure legend, table legend, main text, or Methods section.

n/a	Confirmed	
☐	☒	The exact sample size (*n*) for each experimental group/condition, given as a discrete number and unit of measurement
☐	☒	A statement on whether measurements were taken from distinct samples or whether the same sample was measured repeatedly
☐	☒	The statistical test(s) used AND whether they are one- or two-sided<br>*Only common tests should be described solely by name; describe more complex techniques in the Methods section.*
☒	☐	A description of all covariates tested
☐	☒	A description of any assumptions or corrections, such as tests of normality and adjustment for multiple comparisons
☐	☒	A full description of the statistical parameters including central tendency (e.g. means) or other basic estimates (e.g. regression coefficient) AND variation (e.g. standard deviation) or associated estimates of uncertainty (e.g. confidence intervals)
☐	☒	For null hypothesis testing, the test statistic (e.g. *F*, *t*, *r*) with confidence intervals, effect sizes, degrees of freedom and *P* value noted<br>*Give P values as exact values whenever suitable.*
☒	☐	For Bayesian analysis, information on the choice of priors and Markov chain Monte Carlo settings
☒	☐	For hierarchical and complex designs, identification of the appropriate level for tests and full reporting of outcomes
☐	☒	Estimates of effect sizes (e.g. Cohen's *d*, Pearson's *r*), indicating how they were calculated

Our web collection on statistics for biologists contains articles on many of the points above.

Software and code

Policy information about availability of computer code

Data collection	Image data collection was performed employing Tanon App for Biology Software (Version 1.0.0000), Nikon e200 (Capture2.2), Canon EOS REBEL T1i, EVOS M5000, and Caliper Spectrum IVIS Imaging (Xenogen, US,Version 4.0).<br>The Luminescence was quantified by an automated microplate spectrophotometer (BioTek Synergy H1, USA).<br>Flow cytometry was performed employing BECKMAN COULTER CytoFLEX S (Cyt Expert 2.3).<br>qPCR was performed employing Bio-Rad CFX connect (Version 3.1) or qTOWER 3.<br>RNA-seq, scRNA-seq, ATAC-seq, CUT&Tag, eSPAN and ChIP-seq libraries were sequenced on Illumina NovaSeq 6000 Sequencing system.
Data analysis	Data analysis was performed in R (version 4.03). All FASTQ data was trimmed using Trimmomatic (v.0.39).<br>For RNA-seq, squencing reads were aligned to the reference genome hg38, and gene annotations were added for Homo sapiens, using the STAR (v.2.7.7a) software with default settings. Read counting and differential gene expression analysis were carried out using Cufflinks (v.2.2.1). Functional enrichment analysis for differentially expressed genes was performed using the topGO (v.2.40.0) R package and MetaScape 3.5.<br>For CUT&Tag, ATAC-seq and ChIP-seq, sequence reads were mapped back to reference genome hg38 using bowtie2 (v.2.4.2). Only consistent pair-end reads were chosen for further analysis. Uniquely aligned reads were extracted using SAMtools (Version 1.7). Peaks were called using MACS2 (version 2.2.7.1) and SICER (version 1.0). For each data source, peaks were called using all samples from all replicates combined. For visualization, bigWig files and count matrices in bins around peak summits were produced using Bedtools (v.2.29.2) and bedGraphToBigWig (v.4). The DiffBind R package (v.3.0) was used to identify peaks that differed between MCM2-2A mutant and WT cells. 3D PCA plots were visualized using the rgl R package (v.0.105.13). The Homer suite (v.2.0) was used to annotate peaks.<br>Signal quantification was implemented mainly using custom Perl (v.5.26.2) scripts.<br>Genome transforming from hg19 to hg38 was performed with the UCSC liftOver tool (http://genome.ucsc.edu/cgi-bin/hgLiftOver).<br>Hi-C matrix data were processed by Juicer tools (v2.20.00).

Super enhancers were identified using ROSE (v1) based on the H3K27ac ranking signal.
For scRNA-seq, data was preprocessed by kallisto-bustools (v.0.46.0) workflow, and further analysed by Seurat (v.4.0.3) R packages. Seurat (v.4.0.3) combined with the ggplot2 R package (v2_3.3.5) were used to visualize the expression of selected genes across clusters and samples.
LARRY pipeline was adopted to call lineage clones (Klein AM, 2020, Science). We used custom python (v.3.8) scripts to extract all cell barcodes, UMIs, and lineage barcodes. Enrichment pathway analysis was implemented in the GSVA R package (v1.38.2). Cancer hallmark and GO biological pathway were exported using the msigdbr R package (v7.4.1)
Breast cancer subtype identification was implemented by the supervised risk PAM50 predictor previously provided (Bernard PS, 2009, J Clin Oncol).
eSPAN anaylsis was implemented as described in the publication, Watson and Crick strands were merged and partition or RFD was calculated using public R scripts provided by the eSPAN pipeline (Zhiming Li, Nat Protoc, 2021).
The number of EdU-positive cells and all the cells (marked by DAPI) and quantification analysis of Western blot band intensity were determined with ImageJ (v150) software.
TBtools-II (v1.115) was used for heatmap analysis and venn plot.
Flow cytometry was analyzed using the Cyt Expert 2.3.

Code availability. We have made use of publicly available software and tools. The published code of normalization and PAM50 scripts are available with the following link: https://static-content.springer.com/esm/art%3A10.1186%2Fs12864-019-5849-0/MediaObjects/12864_2019_5849_MOESM6_ESM.zip. The published pipeline used to calculate bias via eSPAN analysis is available with the following link: https://github.com/clouds-drift/eSPAN-bias. The published scripts used to call lineage barcodes are available with the following link: https://github.com/AllonKleinLab/LARRY. All other codes used to generate the analysis have been placed in Zenodo through the following link: https://zenodo.org/record/7927636.

For manuscripts utilizing custom algorithms or software that are central to the research but not yet described in published literature, software must be made available to editors and reviewers. We strongly encourage code deposition in a community repository (e.g. GitHub). See the Nature Portfolio guidelines for submitting code & software for further information.

Data

Policy information about availability of data

All manuscripts must include a data availability statement. This statement should provide the following information, where applicable:

- Accession codes, unique identifiers, or web links for publicly available datasets
- A description of any restrictions on data availability
- For clinical datasets or third party data, please ensure that the statement adheres to our policy

Data availability. The deep sequencing data generated in this study have been deposited in the Gene Expression Omnibus (GEO) database under accession code GSE201262 (https://www.ncbi.nlm.nih.gov/geo/query/acc.cgi?acc=GSE201262), including all the raw data and processed data. The Hi-C processed data of WT MCF-7 and T47D cells used in this study are available in the GEO database under accession code GSM1631185 (https://www.ncbi.nlm.nih.gov/geo/query/acc.cgi?acc=GSM1631185) and ENCODE database under accession code ENCSR549MGQ (https://www.encodeproject.org/experiments/ENCSR549MGQ/), respectively (detailed in Methods section). The gene sets related to breast cancer invasion, metastasis and progression in patients was obtained from Molecular Signatures Database (https://www.gsea-msigdb.org/gsea/msigdb/genesets.jsp?collection=CGP). Cancer hallmark datasets were obtained from Molecular Signatures Database (https://www.gsea-msigdb.org/gsea/msigdb/human/genesets.jsp?collection=H). Cancer functional state score of breast cancer single cells was calculated based on the data sets from CancerSEA (http://biocc.hrbmu.edu.cn/CancerSEA/). Source data are provided with this paper. Large source data sheets were deposited in Zenodo database under accession code 7927636 (https://zenodo.org/record/7927636).

Human research participants

Policy information about studies involving human research participants and Sex and Gender in Research.

Reporting on sex and gender	N/A
Population characteristics	N/A
Recruitment	N/A
Ethics oversight	N/A

Note that full information on the approval of the study protocol must also be provided in the manuscript.

Field-specific reporting

Please select the one below that is the best fit for your research. If you are not sure, read the appropriate sections before making your selection.

☒ Life sciences ☐ Behavioural & social sciences ☐ Ecological, evolutionary & environmental sciences

For a reference copy of the document with all sections, see nature.com/documents/nr-reporting-summary-flat.pdf

Life sciences study design

All studies must disclose on these points even when the disclosure is negative.

Sample size	No sample size calculation was performed to predetermine sample size.<br>For bulk RNA-seq and CUT&Tag, at least 2 biological replicates (2 independent clones) were indicated based on previously published work and comparable studies as standard for this field of research (Kunihiko Hinohara & Franziska Michor, 2018, Cancer Cell; Iannis Aifantis & Cigall Kadoch, 2023, Molecular Cell). Results were validated across 3 different cell lines.<br>For western blot, 2 biological replicates (or 2 independent clones) were preformed across 3 different cell lines to validated the results.<br>For in vivo studies, 5-10 biological replicates in each group were performed based on previously published comparable studies as standard for this field of research (Mark A. Dawson, 2021, Nature; Eugen Dhimolea & Constantine S. Mitsiades, 2021, Cancer Cell).<br>For 3D organoid culture, at least 4 independent experiments in each group were performed based on previously published comparable studies (Kerstin Schneeberger, 2020, Adv. Funct. Mater).<br>For qPCR, at least 3 independent replicates were performed based on our previously published comparable studies (Zhiguo Zhang, 2018, elife).
Data exclusions	For bulk RNA-Seq and CUT&Tag, no data were excluded from the analysis.<br>For scRNA-Seq, cells that did not pass quality control filters (outlined in detail in text) were removed.
Replication	CUT&Tag for H3K27me3, H3K4me3, H3K36me3, H3K9me3 H3K27ac, H3K4me1, H3.3, H2AK119Ub, SUZ12, and RING1B, ATAC-seq, ChIP-seq and bulk RNA-Seq were performed in 2 independent clones of WT and MCM2 mutant MCF-7 cells. Besides, CUT&Tag for H3K27me3 in WT and MCM2 mutant MCF-7 cells was performed twice independently. CUT&Tag for H3K27me3 and H3K4me3, as well as bulk RNA-Seq was performed in 2 replicates for WT and MCM2 mutant HEK293T and T47D cells. CUT&Tag for H3K27me3 was performed in 2 replicates for POLE3 KO MCF-7 cells. PCA analysis indicated the results were consistent and reproducible between CUT&Tag or bulk RNA-Seq replicates.<br>Western blot was repeated 3 times over 2 independent clones in WT and MCM2-2A mutant MCF-7 cells and twice in WT and MCM2 mutant HEK293T and T47D cell lines, as well as in POLE3 KO MCF-7 cells. Results obtained were consistent.<br>qPCR experiments were performed in 3 replicates or more independently as described in associated figure legends. Results obtained were consistent.<br>To measure the size of tumors and analyze the survival time of mice bearing tumors, xenograft assay was performed twice. The results obtained were consistent. The histopathologic analysis for lung metastasis and primary tumor was assessed in 8 mice bearing WT and MCM2-2A MCF-7 tumors.<br>3D organoid culture was performed in 4 independent experiments in each genotype in MCF-7 and T47D cell lines. Besides, 3D organoid culture for growth curve and organoids' PI/Hoechst staining assays were repeated twice in WT and MCM2-2A MCF-7 cells, and the results obtained were consistent.
Randomization	Mice were allocated randomly to bear the WT or MCM2-2A mutant tumors. Other experiments were not relevant as the cells are grouped based on their genotype.
Blinding	The investigators were not blinded to group allocation during data collection and analysis. Because, blinding was not required as each experiment was designed to give an explicit outcome which is independent on the judgement of the researchers.

Reporting for specific materials, systems and methods

We require information from authors about some types of materials, experimental systems and methods used in many studies. Here, indicate whether each material, system or method listed is relevant to your study. If you are not sure if a list item applies to your research, read the appropriate section before selecting a response.

Materials & experimental systems

n/a	Involved in the study	
☐	☒	Antibodies
☐	☒	Eukaryotic cell lines
☒	☐	Palaeontology and archaeology
☐	☒	Animals and other organisms
☒	☐	Clinical data
☒	☐	Dual use research of concern

Methods

n/a	Involved in the study	
☐	☒	ChIP-seq
☐	☒	Flow cytometry
☒	☐	MRI-based neuroimaging

Antibodies

Antibodies used	For CUT&Tag, antibodies against H3K36me3 (Active Motif, cat.# 61021), H3K27me3 (Cell Signaling Technology, Cat.# 9733), H3K27ac (Cell Signaling Technology, Cat.# 8173), H3K9me3 (Abcam, Cat.# ab8898), H3K4me3 (Active Motif, Cat.# 39159), H3K4me1 (Cell Signaling Technology, Cat.# 5326), H3.3 (proteintech, Cat.# 13754-1-ap), SUZ12 (Cell Signaling Technology Cat.# 3737), H2AK119Ub (Cell Signaling Technology Cat.# 8240), and RING1B (Cell Signaling Technology Cat.# 5694) were used. And second antibodies rabbit-anti-mice IgG H&L (Abcam, Cat.# ab46540, Lot GR386449-4) and Donkey-anti-rabbit IgG H&L (sigma, Cat.# SAB 3700932, Lot RI34105) were used.<br>For Western blotting analysis, antibodies against MCM2 (cell Signaling Technology, Cat #3619), β-Actin (Beyotime, Cat.#AF0003), POLE3 (Bethyl, Cat.#A301245A), H3K36me3 (Active Motif, cat.# 61021), H3K27me3 (Cell Signaling Technology, Cat.# 9733), H3K27ac

(Cell Signaling Technology, Cat.# 8173), H3K9me3 (Abcam, Cat.# ab8898), H3K4me3 (Active Motif, Cat.# 39159), H3K4me1 (Cell Signaling Technology, Cat.# 5326), H3.3 (proteintech, Cat.# 13754-1-ap) and H3 (Abcam, Cat.# ab1791) were used.
For Western blotting analysis, HRP-conjugated goat anti-mice (Beyotime, Cat.# A0216) and HRP-conjugated goat anti-rabbit (Beyotime, Cat.# A0208) antibody were used.
For ChIP-seq, EZH2 antibody (Cell Signaling Technology, Cat.# 5246) was used.
For eSPAN, antibodies against H3K36me3 (Active Motif, cat.# 61021) and second antibodies rabbit-anti-mice IgG H&L (Abcam, Cat.# ab46540) were used.

Validation

Anti-H3K36me3 (Active Motif, cat.# 61021) were reported to be applicant for CUT&Tag and eSPAN (Zhiguo Zhang S, Sci Adv. 2020).
Anti-H3K27me3 (Cell Signaling Technology, Cat #9733) and Anti-H3K4me3 (Active Motif, Cat #39159) were reported to be applicant for CUT&Tag (Kaya-Okur HS, Nat Commun. 2019, Henikoff S, Elife. 2020 and Kaya-Okur HS, Nat Protoc. 2020).
Anti-H3K9me3 (Abcam, Cat ab8898) were reported to be applicant for CUT&Tag (Henikoff S, Elife. 2020).
EZH2 antibody (Cell Signaling Technology, Cat.# 5246) were reported to be applicant for ChIP-seq (GSM2486302).
Anti-H3.3 (Proteintech, Cat 13754-1-ap) CUT&Tag datasets were validated in this study by comparison to published ChIP-seq datasets (GSE95977).
Anti-H3K27ac (Cell Signaling Technology, Cat.# 8173) CUT&Tag datasets were validated in this study by comparison to published ChIP-seq datasets (GSE96352; GSM945854).
H3K4me1 (Cell Signaling Technology, Cat.# 5326) CUT&Tag datasets were validated in this study by comparison to published ChIP-seq datasets (GSE86714).
SUZ12 (Cell Signaling Technology Cat.# 3737) CUT&Tag datasets were validated in this study by comparison to published ChIP-seq datasets (GSE105981).
RING1B (Cell Signaling Technology Cat.# 5694) CUT&Tag datasets were validated in this study by comparison to published ChIP-seq datasets (GSE107176).
H2AK119Ub (Cell Signaling Technology Cat.# 8240) CUT&Tag datasets were validated in this study by comparison to published ChIP-seq datasets (GSE107176).
H3 (Abcam, Cat.# ab1791) was suitable for western blotting analysis of human cells according to the manufacturer's instructions (https://www.abcam.cn/products/primary-antibodies/histone-h3-antibody-nuclear-marker-and-chip-grade-ab1791.html), and was reported to be applicant for western blotting analysis in human cell lines (Dong Fang & Song-Qiang Xie,2020, Cancer science, PMID: 33164305).
MCM2 (cell Signaling Technology, Cat #3619) was suitable for western blotting analysis of human cells according to the manufacturer's instructions (https://www.cellsignal.cn/products/primary-antibodies/mcm2-d7g11-xp-rabbit-mab/3619?site-search-type=Products&N=4294956287&Ntt=3619&fromPage=plp&_requestid=931661), and was reported to be applicant for western blotting analysis (Holger Bastians, 2022, Cell Rep, PMID: 36516748).
β-Actin (Beyotime, Cat.# AF0003) was suitable for western blotting analysis of human cells according to the manufacturer's instructions (https://www.beyotime.com/product/AF0003.htm), and was reported to be applicant for western blotting analysis in human cell lines (Yongqian Shu & Tongpeng Xu, 2020, Mol Cancer, PMID: 32600329).
POLE3 (Bethyl, Cat.#A301245A) was suitable for western blotting analysis of human cells according to the manufacturer's instructions (https://www.thermofisher.cn/cn/zh/antibody/product/PolE3-p17-Antibody-Polyclonal/A301-245A), and was reported to be applicant for western blotting analysis (Zhiguo Zhang, 2020, Science Advances, PMID: 32923642).

Eukaryotic cell lines

Policy information about cell lines and Sex and Gender in Research

Cell line source(s)

Regular MCF-7, T47D and 293T cell lines were obtained from ATCC. Mutation cell lines were generated by introducing mutations into MCF-7, T47D and 293T cells. POLE3 KO cell lines were generated by introducing indels into MCF-7 cells.

Authentication

MCF-7, T47D and 293T cell lines were authenticated by transcriptional profiling. The transcriptome of our MCF-7 cells is highly similar to that of published MCF-7 datasets on GSE128460 (Bi M, Nat Cell Biol. 2020). The transcriptome of our T47D cells is highly similar to that of published T47D datasets on GSE179666 (Zaurin R,Nucleic Acids Res. 2021). The transcriptome of our 293T cells is highly similar to that of published 293T datasets on GSE196384 (Shin HR, Science. 2022)
Genome editing lines were validated by genomic PCR with specific primer sets and Sanger sequencing.

Mycoplasma contamination

All cell lines were routinely tested negative for mycoplasma contamination.

Commonly misidentified lines
(See ICLAC register)

None.

Animals and other research organisms

Policy information about studies involving animals; ARRIVE guidelines recommended for reporting animal research, and Sex and Gender in Research

Laboratory animals

Five- to six-week-old female NOD/ShiLtJGpt-Prkdcem26Cd52Il2rgem26Cd22/Gpt (NCG) mice were purchased from GemPharmatech Co., Ltd. All mice were housed in the specific pathogen-free (SPF) room under controlled temperature (20-26℃) and humidity (40-70%) conditions with 12/12h light/dark cycle.

Wild animals

This study did not use wild animals.

Reporting on sex

Female mice were used in this study, because MCF-7 is a cell line from breast cancer, which highly occurs in female. And female mice were routinely used in breast cancer related researches (Bi M., Nat Cell Biol. 2020; Hinohara, K., Cancer Cell. 2018).

Field-collected samples

This study did not involve field-collected samples.

Ethics oversight	All experiments reported herein was approved by Institutional Animal Care and Use Committee (IACUC) at Shenzhen Institutes of Advanced Technology, Chinese Academy of Sciences (SIAT-IACUC-200319-HCS-TCC-A1148).

Note that full information on the approval of the study protocol must also be provided in the manuscript.

ChIP-seq

Data deposition

☒ Confirm that both raw and final processed data have been deposited in a public database such as GEO.

☒ Confirm that you have deposited or provided access to graph files (e.g. BED files) for the called peaks.

Data access links<br>*May remain private before publication.*	DataBank Accession: GEO<br>Accession ID: GSE201262<br>Databank URL: https://www.ncbi.nlm.nih.gov/geo/query/acc.cgi?&acc=GSE201262
Files in database submission	Files entitled with "WT MCF-7 EZH2 ChIP-seq repeat 1", "WT MCF-7 EZH2 ChIP-seq repeat 2", "MCM2-2A MCF-7 EZH2 ChIP-seq repeat 1", and "MCM2-2A MCF-7 EZH2 ChIP-seq repeat 2" are ChIP-seq data of EZH2.
Genome browser session<br>(e.g. UCSC)	All bigwig files for ChIP-seq data have been deposited to GEO dataset GSE201262 as processed file.

Methodology

Replicates	WT (n = 2 independent clone lines) and MCM2-2A mutant (n = 2 independent clone lines) cells were used to perform ChIP-seq.
Sequencing depth	20 million pair-end raw reads for each sample with the length of 150bp.
Antibodies	EZH2 antibody (Cell Signaling Technology, Cat.# 5246)
Peak calling parameters	EZH2 peaks were detected using MACS2 (v.2.2.7.1) with a P-value cutoff of 0.01.
Data quality	FASTQC (v0.11.9) is run to check the sequencing quality.
Software	FASTQC (v0.11.9), Trimmomatic (v.0.39), bowtie2 (v.2.4.2), Samtools (v.1.7), MACS2 (v.2.2.7.1), Bedtools (v.2.29.2), bedGraphToBigWig (v.4), and DiffBind R package (v.3.0).

Flow Cytometry

Plots

Confirm that:

☒ The axis labels state the marker and fluorochrome used (e.g. CD4-FITC).

☒ The axis scales are clearly visible. Include numbers along axes only for bottom left plot of group (a 'group' is an analysis of identical markers).

☒ All plots are contour plots with outliers or pseudocolor plots.

☒ A numerical value for number of cells or percentage (with statistics) is provided.

Methodology

Sample preparation	To evaluate the cell proliferation, the organoids were treated with Edu at 50 µM for 5 h. The organoids were trypsinized into single cells and fixed with 70% ethanol in PBS at -20°C overnight. The cells were pellet and resuspended in PBS containing 0.2% triton X-100. After 2 washes with PBS, Click-it reaction was performed according to the manufacturer's instructions (Beyotime, Cat.#C0081S). After 2 washes with PBS, the cells were treated with PI (1:500) and RNase A (20µg/ml) for 30 min at room temperature, protected from light. Samples were stored at 4 °C and protected from light until analysis by flow cytometry (BECKMAN COULTER CytoFLEX S).<br>For cell sorting, GFP+ MCF-7 cells were washed with PBS, collected by trypsinization, filtered using a 40 µm filter, then sorted for EGFP positive cells .
Instrument	BECKMAN COULTER CytoFLEX S was used for FACS analysis. BD FACSAria III was used for cell sorting.
Software	Cyt Expert 2.3
Cell population abundance	At least 1×10^5 EGFP positive cells were sorted from each MCF-7 line.
Gating strategy	For quantification of Edu positive cells, intact cells were selected by comparison of cell size (FSC-A) and cell granularity (SSC-A). Single cells were detected by analyzing the PI cell area versus the PI height, singlets fall on the diagonal between the two axis. Compensation was performed using the appropriate negative control samples. Percentage of Edu + cells were quantified.

For EGFP positive cell sorting, the EGFP threshold for gating was established using the positive control samples. A negative control sample confirmed this gating strategy. The exact same gate was applied to all experimental samples.

☒ Tick this box to confirm that a figure exemplifying the gating strategy is provided in the Supplementary Information.
